# Anatomie und Physiologie des Lymphgefäßsystems: Was man in der Klinik wissen sollte

**DOI:** 10.1007/s00117-025-01432-2

**Published:** 2025-03-22

**Authors:** Erich Brenner

**Affiliations:** Institut für Klinisch-Funktionelle Anatomie, Medizinische Universität Innsbruck, 6020 Innsbruck, Österreich

**Keywords:** Initiale Lymphgefäße, Präkollektoren, Kollektoren, Lymphknoten, Lymphfluss, Initial lymph vessels, Precollectors, Collectors, Lymph nodes, Lymph flow

## Abstract

Die Lymphgefäße sind ein unidirektionales, zentripetales System, das interstitielle Flüssigkeit aus Geweben und Organen sammelt und in das Venensystem leitet. Die Lymphgefäße lassen sich in 4 Typen gliedern: initiale Lymphgefäße, Präkollektoren, Kollektoren und Lymphstämme. Initiale Lymphgefäße formen Netzwerke mit größeren Maschen als Blutkapillaren und sind mit speziellem Endothel ausgekleidet. Sie weisen interendotheliale Öffnungen auf, welche den Austausch mit dem umgebenden Gewebe ermöglichen. Diese Gefäße sind zudem mit charakteristischen Markern wie VEGFR‑3, Podoplanin und LYVE‑1 ausgestattet. Präkollektoren verfügen über glatte Muskelzellen, die noch unregelmäßig angeordnet sind, und parietale Klappen, die den zentripetalen Lymphfluss fördern. Kollektoren haben eine durchgehende Media aus spiralig angeordneten Muskelfasern, die eine Verengung und Kontraktion der Gefäße ermöglicht. Diese Kontraktion führt zum Transport der Lymphe durch einzelne Lymphangienabschnitte. Lymphstämme bilden den nächsten Abschnitt und münden oft in den Ductus thoracicus, der sich in das Venensystem öffnet. Lymphstämme enthalten zunehmend strukturierte Abschnitte und Klappen zur weiteren Regulation des Lymphflusses.

Lymphknoten sind in den Verlauf der Kollektoren und Stämme eingebaut und dienen als Kontrollstationen für Krankheitserreger und die Regulation des kolloidosmotischen Drucks der Lymphe. Sie sind in Gruppen organisiert und haben eine spezifische Struktur mit Kortex, Medulla und eingelagerten B‑ und T‑Lymphozyten. Das lymphatische Hauptsystem transportiert die Lymphe durch segmentale Verbindungen und Anastomosen. Der Ductus thoracicus mündet in den linken Venenwinkel und verbindet sich mit weiteren Lymphgefäßen. Die Entwicklungsgeschichte und Variationen des Ductus thoracicus führen zu gelegentlichen anatomischen Abweichungen.

## Abstract (en)

The lymphatic vessels are a unidirectional, centripetal system that collects interstitial fluid from tissues and organs, directing it into the venous system. The lymphatic vessels can be categorized into four types: initial lymphatic vessels, precollectors, collectors, and lymphatic trunks. Initial lymphatic vessels comprise interconnected networks characterized by larger mash sizes compared to blood capillaries and are lined with a specialized endothelium. They have interendothelial openings that allow exchange with the surrounding tissue. These vessels also have characteristic markers such as VEGFR‑3, podoplanin, and LYVE‑1. Precollectors have smooth muscle cells that are still arranged irregularly and parietal valves that promote centripetal lymph flow. Collectors have also valves and a continuous layer of spirally arranged muscle fibers that allow the vessels to pulse and contract. This contraction causes the lymph to be transported from one lymphangion to the next. Lymphatic trunks form the next section and often flow into the thoracic duct, which opens into the venous system. Lymph nodes are situated along the course of the collectors and trunks and serve as filter centers to intercept pathogens and increase the colloid osmotic pressure of the lymph. They are organized into groups with a specific structure with cortex, medulla, and embedded B and T lymphocytes. The central lymphatic system transports lymph through segmental connections and anastomoses. The thoracic duct empties into the left venous angle and collects also other lymphatic truncs. The thoracic duct’s developmental history and variations occasionally lead to anatomical anomalies.

Das Lymphsystem: Wächter des Körpers und Meister der Flüssigkeitsregulation. Erfahren Sie, wie ein fein verzweigtes Netz aus Gefäßen und Knoten unser Immunsystem schützt, Gewebe entwässert und lebenswichtige Prozesse steuert. Ein faszinierender Einblick in die unsichtbare Kraft, die Gesundheit und Heilung fördert – unverzichtbar für Medizin, Therapie und Forschung!

## Die Lymphgefäße

Lymphgefäße, Saugadern, stellen ein unidirektionales, zentripetales Gefäßsystem dar, dass die interstitielle Flüssigkeit verschiedenster Gewebe und Organe sammelt und direkt oder indirekt in das Venensystem zurückführt. Das zentrale Nervensystem, Knochen und das Auge haben jedoch keine Lymphgefäße [1].

Die Lymphgefäße lassen sich einteilen in:

- Initiale Lymphgefäße
- Präkollektoren
- Kollektoren
- Lymphstämme

### Initiale Lymphgefäße

Die initialen Lymphgefäße bilden in der Regel zwei- oder dreidimensionale Netze, deren Maschengröße deutlich über jener der (Blut‑)Kapillaren liegt. Diese Netzwerke umgeben die einzelnen Organe bzw. finden sich in der Kutis. Eine Ausnahme davon bilden die absorbierenden Lymphgefäße in den Darmzotten; hier befindet sich in jeder Darmzotte ein einzelnes, blind beginnendes initiales Lymphgefäß. Erst in der eigentlichen Mukosa bildet sich das charakteristische Netzwerk.

**Abb. 1:**
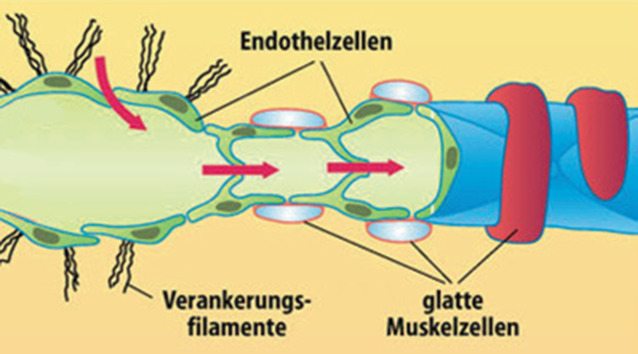
Initiales Lymphgefäß (*links*) und Präkollektor (*rechts*). (Aus [7] Mit freundl. Genehmigung von Springer Medizin Verlag Heidelberg)

Initiale Lymphgefäße sind von einem speziellen Endothel ausgekleidet, das von einem subendothelialen Faserfilz umgeben ist. In Gegensatz zu Blutkapillaren schließen die Lymphendothelzellen jedoch das Lumen nicht vollständig ab. Vielmehr wechseln entlang der Zellgrenzen langgestreckte Zonen, in denen benachbarte Lymphendothelzellen durch Schlussleisten (Zonulae occludentes oder „tight junctions“) fest miteinander verbunden sind, mit kürzeren Abschnitten, in denen eine offene Verbindung zum umgebenden Interstitium besteht (interendotheliale Öffnungen oder „open junctions“; [2]). Lymphendothelzellen lassen sich auch immunhistochemisch gut von Blutendothelzellen unterscheiden, denn sie exprimieren eine Vielzahl von charakteristischen Markern: VEGFR‑3, einen Tyrosinkinase-Rezeptor für VEGF‑C und VEGF‑D, Podoplanin, Prox‑1, und LYVE‑1, einen Hyaluronan-Rezeptor [3]. LYVE‑1 ist vornehmlich an den freien Rändern der interendothelialen Öffnungen situiert. Der subendotheliale Faserfilz ist deutlich lockerer als die Basalmembran von Blutkapillaren. Zudem besitzen initiale Lymphgefäße Ankerfilamente aus elastischen Fasern, die an der äußeren Zellwand der Lymphendothelzellen angeheftet sind und in die Umgebung ausstrahlen [4]. Die in die initialen Lymphgefäße aufgenommene Flüssigkeit, die Lymphe, kann in den initialen Lymphgefäßnetzwerken in alle Richtungen fließen (Abb. 1).

#### Merke.

Die initialen Lymphgefäße bilden grundsätzlich Netzwerke aus.

### Präkollektoren

In der Wand von Präkollektoren treten erstmals glatte Muskelzellen und parietale Klappen auf. Die glatten Muskelzellen sind noch unregelmäßig und diskontinuierlich angeordnet. Da die Media noch nicht vollständig ausgebildet ist, können auch Präkollektoren interstitielle Flüssigkeit aufnehmen. Die Klappen werden vom Endothel gebildet und bedingen erstmals einen zentripetal gerichteten Flüssigkeitsstrom.

#### Merke.

Präkollektoren besitzen bereits einen zentripetal gerichteten Lymphstrom, sind aber noch selbst zur Aufnahme interstitieller Flüssigkeit fähig.

### Kollektoren

Die Kollektoren besitzen eine durchgehende Media aus gegenläufig spiralig angeordneten glatten Muskelfasern [5]. Diese Anordnung erlaubt sowohl eine Längen- wie auch Weitenverengerung. Die Kollektoren bestehen aus hintereinandergeschalteten Lymphangien, die von den parietalen Lymphklappen unterteilt werden. Jedes Lymphangion agiert dabei grundsätzlich isoliert; der wichtigste Trigger für eine Kontraktion ist der Füllungszustand des jeweiligen Lymphangions. Durch die Kontraktion eines Lymphangions wird der Inhalt, die Lymphe, in das nachfolgende Lymphangion weitergeschoben, wodurch dieses gedehnt wird. Dadurch wird dann mit leichter Verzögerung ebenfalls eine Kontraktion ausgelöst und die Lymphe weitertransportiert.

In den Verlauf von Kollektoren sind in der Regel an charakteristischen Stellen Lymphknoten eingebaut. Die tiefen, unter der Hüllfaszie liegenden Kollektoren verlaufen zumeist eng an die jeweiligen Arterien geschmiegt.

### Lymphstämme

Im Stamm vereinigen sich die Lymphkollektoren zu den noch weiteren Lymphstämmen, deren größter, der Ductus thoracicus, üblicherweise in den linken Venenwinkel zwischen V. brachiocephalica sinistra und V. jugularis interna sinista einmündet. Die Strukturierung in Lymphangien wird zunehmend aufgelöst, wobei dennoch paarige parietale Klappen einzelne Abschnitte unterteilen.

### Lymphknoten

In den Verlauf der Kollektoren und Lymphstämme sind Lymphknoten eingebaut. Diese finden sich überwiegend an mehr oder weniger genau definierten Stellen wie der Achselhöhle oder der Leiste. Sie bilden dabei genau beschriebene Lymphknotengruppen, die aus einem bis zu 10 Lymphknoten bestehen können. So variiert die Gesamtzahl der Lymphknoten im Menschen in weiten Bereichen und liegt zwischen 400 und 800 Lymphknoten.

In der Leistenregion liegen zwei oberflächliche und eine tiefe Lymphknotengruppe. Während die Nodi lymphoidei (Nll.) inguinales profundi zumeist nur aus einigen wenigen Lymphknoten bestehen, die entlang der A. femoralis verlaufen und deren proximaler Lymphknoten (Rosenmüller-Lymphknoten, „node of Cloquet“) direkt im Canalis femoralis liegt, bestehen der horizontale und der vertikale Trakt der Nll. inguinales superficiales üblicherweise jeweils aus mindestens 5 Lymphknoten.

Die Lymphknoten der Achselhöhle werden aus medizinischer Sicht in 3 Level eingeteilt. Level 1 umfasst die lateral und kaudal des M. pectoralis minor liegenden Lymphknoten. Die Lymphknoten des Level II liegen direkt unter dem M. pectoralis minor und befinden sich im Zentrum der Achselhöhle. Der Level III umfasst jene Lymphknoten, die medial und kranial des M. pectoralis minor liegen.

Im Bauchraum liegen die peripheren viszeralen Lymphknoten meist unmittelbar an den jeweiligen Viszeralorganen, gefolgt zumeist von einer weiteren Lymphknotenstation an den Verzweigungsstellen der größeren Arterien und einer Lymphknotenstation an den Gekrösewurzeln.

**Abb. 2:**
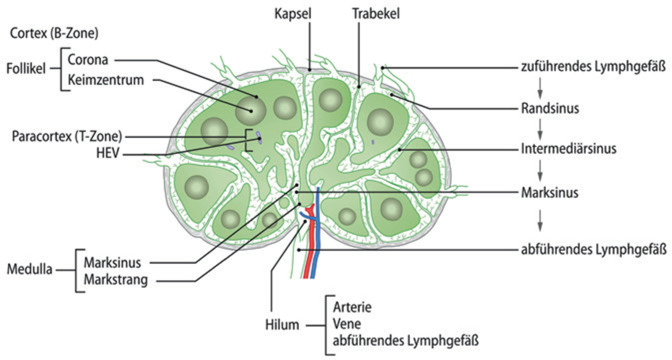
Aufbau und Struktur des Lymphknotens. (Aus [8], mit freundl. Genehmigung von Springer Medizin Verlag Heidelberg)

Lymphknoten besitzen eine relativ straffe Bindegewebekapsel aus der Trabekel in das Innere des Lymphknotens vordringen (Abb. 2). Dadurch wird der Lymphknoten in mehrere Läppchen – unvollständig – unterteilt. Das Innere wird von einem retikulären Bindegewebe ausgefüllt. In dieses sind die Lymphozyten eingelagert, im Kortex finden sich hauptsächlich B‑Lymphozyten, die in kugeligen Lymphfollikeln angeordnet sind. In der parakortikalen Zone am Übergang zwischen Rinde und Mark finden sich überwiegend T‑Zellen. In der Medulla finden sich sowohl B‑ und T‑Zellen als auch Makrophagen. Jedes Lymphknotensegment besitzt zumindest ein zuführendes Lymphgefäß, Vas afferens, zumeist ein kleinerer Kollektor. Die Lymphe verteilt sich über den Randsinus, den entlang der Trabekel verlaufenden Intermediärsinus und den Marksinus. Aus dem Hilum entspringen dann ein oder zwei abführende Lymphgefäße, Vasa efferentes. Diese werden von ableitenden Venen begleitet, wohingegen eine Arterie in den Lymphknoten eintritt.

Die Lymphknoten haben mehrere Aufgaben. Zum einen dienen sie im Immunsystem als wichtige Kontrollstation für (eingedrungene) Krankheitserreger und andere Antigene. Zum anderen kontrollieren und regulieren sie den kolloidosmotischen Druck der Lymphe indem sie zumeist Wasser aus der Lymphe in die Blutgefäße resorbieren und so den kolloidosmotischen Druck der Lymphe dem Plasma annähern. In der Regel werden etwa 50 % der Flüssigkeit aus der Lymphe resorbiert.

## Die tiefen Lymphstämme des Stammes

**Abb. 3:**
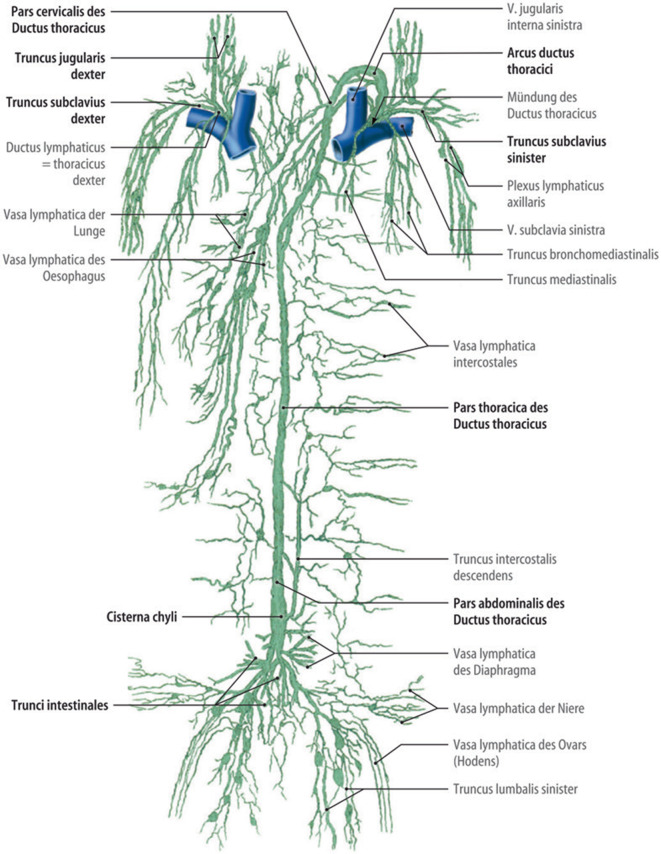
Übersicht über die Hauptlymphstämme. (Aus [9]. Mit freundl. Genehmigung von Springer Medizin Verlag Heidelberg)

Die parietalen tiefen Lymphstämme des Stammes sind grundsätzlich bilateral symmetrisch aufgebaut, die viszeralen Stämme wie auch die Arterien und Venen einfach. Das Ziel der Lymphabflüsse ist der jeweilige Venenwinkel, also die Vereinigung von V. subclavia und V. jugularis interna zur V. brachiocephalica (Abb. 3).

Im rechten Venenwinkel münden üblicherweise die 3 Lymphstämme von Arm (Truncus subclavius dexter), Kopf und Hals (Truncus jugularis dexter) sowie dem Brustraum (Truncus bronchiomediastinalis dexter). Diese vereinigen sich meist unmittelbar vor ihrer Einmündung zum Ductus lymphaticus dexter.

Im linken Venenwinkel, klinisch oft als Terminus bezeichnet, münden die Trunci subclavius, jugularis und bronchomediastinalis sinister. Hinzu kommt der asymmetrische Ductus thoracicus, der von dorsal durch das Trigonum scalenovertebrale nach ventral zieht. Auch hier kommt es üblicherweise zu einer gemeinsamen Stammbildung, isolierte Einmündungen vor allem des Truncus jugularis sinister kommen jedoch öfters vor.

Der Ductus thoracicus ist ein entwicklungsgeschichtliches Überbleibsel des ursprünglich symmetrisch angelegten Lymphabflusses aus der unteren Körperhälfte. Die linken und rechten Trunci iliaci communes setzen sich nach kranial in die beiden Trunci lumbales fort. Sie werden dabei durch zarte transversale Anastomosen miteinander verbunden. Auf Höhe des ersten oder zweiten Lendenwirbelkörpers, kaudal der Nierengefäße und rechts der Aorta, mündet der Truncus interstinalis in den Truncus lumbalis dexter ein und bildet mit diesem mitunter eine sackartige Erweiterung, die Cisterna chyli. Die Angaben über die Häufigkeit einer sackartigen Erweiterung als Cisterna chyli schwanken in weiten Bereichen, es kann aber ungefähr eine Häufigkeit von etwa 50 % angenommen werden [6]. Auch Länge und Durchmesser werden sehr unterschiedlich angegeben und schwanken von 1–4 cm in der Länge und 0,4–3 cm im Durchmesser [6]. Auf etwa gleicher Höhe mündet der Truncus lumbalis sinister über eine der vorher erwähnten Queranastomosen ein, entweder direkt in die Cisterna chyli, aber auch etwas kaudal oder kranial davon. Kranial der Cisterna chyli verschwindet der der Truncus lumbalis sinister üblicherweise und nur der Truncus lumbalis dexter setzt sich als Ductus thoracicus (dexter) nach kranial fort. Der Ductus thoracicus passiert das Zwerchfell im Hiatus aorticus zwischen Aorta und V. azygos meist direkt an der Wirbelsäule.

**Abb. 4:**
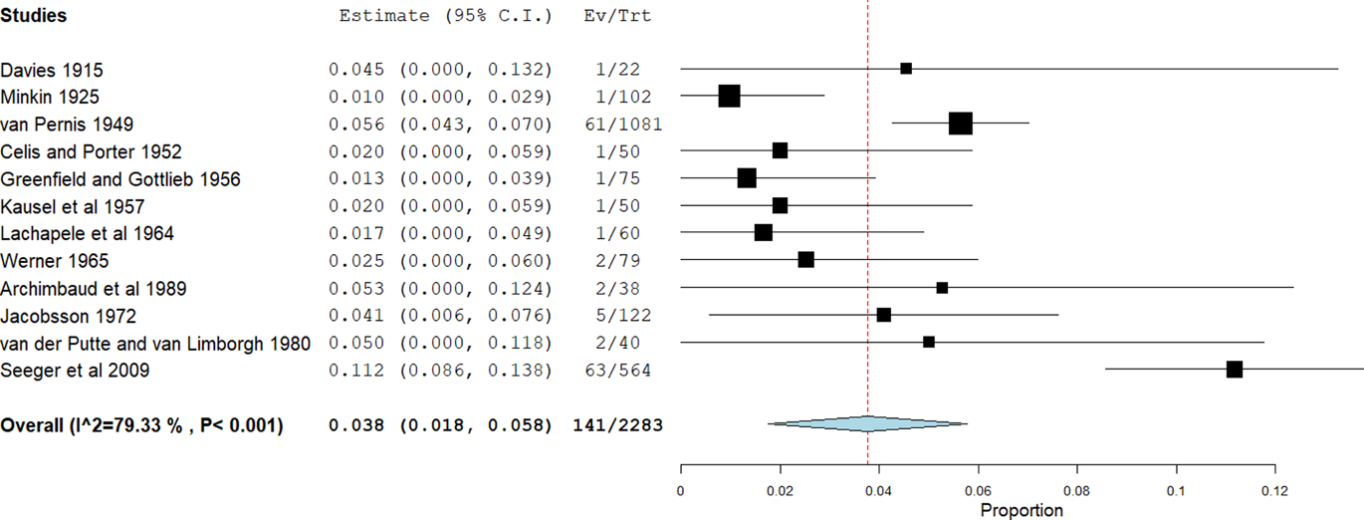
Prävalenz eines Ductus thoracicus dexter. (Aus [6])

Auf Höhe des 6. oder 5. Brustwirbels wechselt der Ductus thoracicus jedoch die Seite. Entwicklungsgeschichtlich endet auf dieser Höhe der rechte aufsteigende Lymphstamm und mündet über die entsprechende transversale Anastomose in den nun wieder kräftig ausgebildeten linken aufsteigenden Lymphstamm. Durch diese Entwicklung kann es auch zu Doppelungen oder Schleifenbildungen des Ductus thoracicus kommen. In etwa 4 % der Fälle (141 von 2283) fehlt jedoch der Seitenwechsel und der Ductus thoracicus mündet dann in den rechten Venenwinkel (Abb. 4), oder sehr selten können sogar ein linker und ein rechter Ductus thoracicus in die entsprechenden Venenwinkel einmünden [6].

Der Ductus thoracicus nimmt die segmentalen interkostalen und paraaortalen Lymphgefäße auf, manchmal auch solche aus dem hinteren Mediastinum.

## Fazit für die Praxis

- Das lymphatische System spielt eine zentrale Rolle in der Flüssigkeitsregulation, Immunüberwachung und dem Transport von Nährstoffen und Abfallstoffen.
- Für die Praxis bedeutet dies, dass ein tiefes Verständnis der Lymphgefäße essenziell ist, insbesondere im Kontext von Erkrankungen wie Lymphödemen, Infektionen und Autoimmunerkrankungen.
- Die Unterscheidung zwischen initialen Lymphgefäßen, Präkollektoren, Kollektoren und Lymphstämmen hilft, gezielte therapeutische Maßnahmen zu planen, z. B. durch Lymphdrainage oder operative Eingriffe.
- Lymphknoten als Filterstationen erfordern bei Entzündungen, Metastasen oder anderen Erkrankungen gezielte Diagnostik und Therapie.
- Eine präzise Kenntnis der Lymphwege fördert präventive, diagnostische und therapeutische Ansätze in der Medizin.
